# Mahaenggamseok-tang, a herbal medicine, for lower respiratory tract infections in pediatric patients: A protocol for systematic review and meta-analysis

**DOI:** 10.1097/MD.0000000000021951

**Published:** 2020-09-04

**Authors:** Aram Jeong, Seung-Bo Yang, Hye-Yoon Lee, Man-Suk Hwang

**Affiliations:** a Department of Pediatrics; b Department of Korean Internal Medicine, College of Korean Medicine, Gachon University, Seongnam; c School of Korean Medicine, Pusan National University; d Department of Rehabilitation Medicine of Korean Medicine, Pusan National University Korean Medicine Hospital, Yangsan, Republic of Korea.

**Keywords:** herbal medicine, lower respiratory tract infection, Mahaenggamseok-tang, pediatric patient

## Abstract

**Background:**

The aim of this study is to assess the clinical evidence for or against Mahaenggamseok-tang (MHGT) as a treatment for lower respiratory tract infections (LRTIs) in pediatric patients.

**Method:**

This systematic review will include randomized clinical trials (RCTs) of MHGT, as a treatment for LRTIs, compared with other therapies such as placebo and western medicine. The search terms will be selected according to the medical subject heading. We will search the following databases for systematic reviews from 2000 to Feb 2020: 5 English databases (The Cochrane Database of Systematic reviews, MEDLINE, Excerpta Medica dataBASE, Allied and Complementary Medicine Database, and Cumulative Index to Nursing and Allied Health Literature), 1 Chinese database, 5 Korean databases (Oriental Medicine Advanced Searching Integrated System, DataBase Periodical Information Academic (DBPIA), Research Information Service System, Korean Studies Information Service System, and National Digital Science Library), and 1 Japanese database (J-Stage). All RCTs of decoctions or alternate forms of MHGT will be included. We will search for all parallel or crossover RCTs without language restrictions. The methodological quality of the RCTs will be assessed using Cochrane risk of bias. Furthermore, the studies will be limited to those performed in children under 16 years of age.

**Results and Conclusions:**

Our systematic review and meta-analysis will provide evidence for MHGT as a treatment for LRTI. The findings can help practitioners and patients recognize more effective and safer therapeutic methods.

**Prospero registration number:**

CRD42020165698

## Abstract 2

Supplemental Digital Content is available in the text

## 1 Introduction

Lower respiratory tract infections (LRTIs) represent one of the most common causes of hospitalization in infants globally.^[1]^ It was the leading cause of death in 2016, accounting for 13.1% of all deaths of children under the age of 5 years.^[1]^ Frequent respiratory infections in early life can result in various respiratory comorbidities such as recurrent wheezing and asthma.^[2]^ Recently, the mortality rate associated with LRTIs has progressively increased owing to the increased appearance of fine dust and new kinds of viruses.^[1]^ Many patients are worried about more severe illnesses, and they have difficulties in caring for their children.^[3]^

The causes of respiratory infections are bacteria (such as mycoplasma, *Staphylococcus aureus*, and *Streptococcus*) and viruses (such as adenovirus, influenza A or B, and the recent novel coronavirus).^[4,5]^ However, it is difficult to distinguish the cause of infection from just clinical symptoms, and antibiotics are often inappropriately (and excessively) used in patients with LRTIs.^[6]^ The use of antibiotics in Korea is the third highest among Organization for Economic Cooperation and Development (OECD) countries; here, 21% of antibiotics are prescribed only for LRTIs.^[7]^ In the US, 70,000 pediatric patients visit the emergency department for antibiotic-related adverse events annually, and 86.1% of these patients have allergic reactions.^[8]^ In addition, previous studies have reported that antibiotics have no major benefits against LRTIs in patients^[9]^; moreover, the overuse of antibiotics can lead to bacterial resistance.^[10,11]^ The treatment of diseases with herbal medicines is regarded as a safer and more effective therapeutic approach than treatment with antibiotics.^[12]^ A study showed that 71.9% of children who had used herbal medicines used them for respiratory diseases.^[13]^ However, research on the effectiveness of herbal medicine in children with lower respiratory tract diseases is insufficient.^[14]^ Therefore, it is necessary to establish evidence for herbal medicines as a treatment for LRTIs.

Mahaenggamseok-tang (MHGT) is a widely prescribed herbal medicine for patients with LRTIs in Asian countries.^[15]^ Recently, it was reported to have activity against severe acute respiratory syndrome coronavirus.^[16]^ The major symptoms of LRTIs are fever, cough, and sputum secretion, which are considered wind-heat syndromes in complementary and alternative medicine. In Korean medicine, MHGT is a representative prescription for alleviating wind-heat invasion in the respiratory tract. It consists of mahuang (*Ephedra sinica*), haengin (*Prunus armeniaca*), gamcho (*Glycyrrhiza uralensis* Fisch.), and seokgo (*Gypsum fibrosum*). Of these components, mahuang has been experimentally used to relieve airway inflammation, and gamcho (licorice) has antiviral effects.^[17]^ Furthermore, seokgo has been reported to have antipyretic effects.^[18]^ In addition, several studies have reported that MHGT exerts anti-inflammatory effects in lung injury by regulating the Nuclear factor-κB (NF-κB) pathway.^[19,20]^

Numerous clinical trials have been performed on MHGT for LRTIs, and they have indicated that it is effective for LRTIs. However, there is no systematic review of such studies. Therefore, we have planned to conduct a review of clinical studies on LRTIs.

## 2 Methods

### 2.1 Data sources

The following electronic databases will be searched from 2000 to February 2020: 5 English databases (The Cochrane Database of Systematic reviews, MEDLINE, Excerpta Medica dataBASE, Allied and Complementary Medicine Database, and Cumulative Index to Nursing and Allied Health Literature), 1 Chinese database (Chinese National Knowledge Infrastructure), 5 Korean databases (Oriental Medicine Advanced Searching Integrated System, DBPIA, Research Information Service System, Korean Studies Information Service System, and National Digital Science Library), and 1 Japanese database (J-Stage). No language restriction will be applied.

Search terms will be mapped to database-defined terms such as medical subheading terms, including [bronchitis or bronchiolitis or pneumonia or lung inflammation or bronchial pneumonia or lower respiratory tract infection] and [Ma Xing Shi Gan or Maxing Ganshi or Maxingshigan or Ma-Xing-Gan-Shi Tang or Mahaenggamseok-tang]. The search terms will be appropriately translated for the different databases. A detailed search strategy is presented in Supplemental Digital Contents (Appendix A and B).

### 2.2 Study selection

#### 2.2.1 Types of studies

We will include all types of randomized controlled trials (RCTs) (eg, parallel and crossover) that assessed the efficacy of MHGT, regardless of blinding, language, and type of reporting. All the included RCTs should have the term “Randomisation (or randomization)” in the methodology. This review will also include sole treatment or complex intervention studies the control treatment was administered equally in both the experimental and control groups (eg, A + B vs B). Dissertations and abstracts will be included if they provide sufficient information.

#### 2.2.2 Types of patients

**Figure 1:**
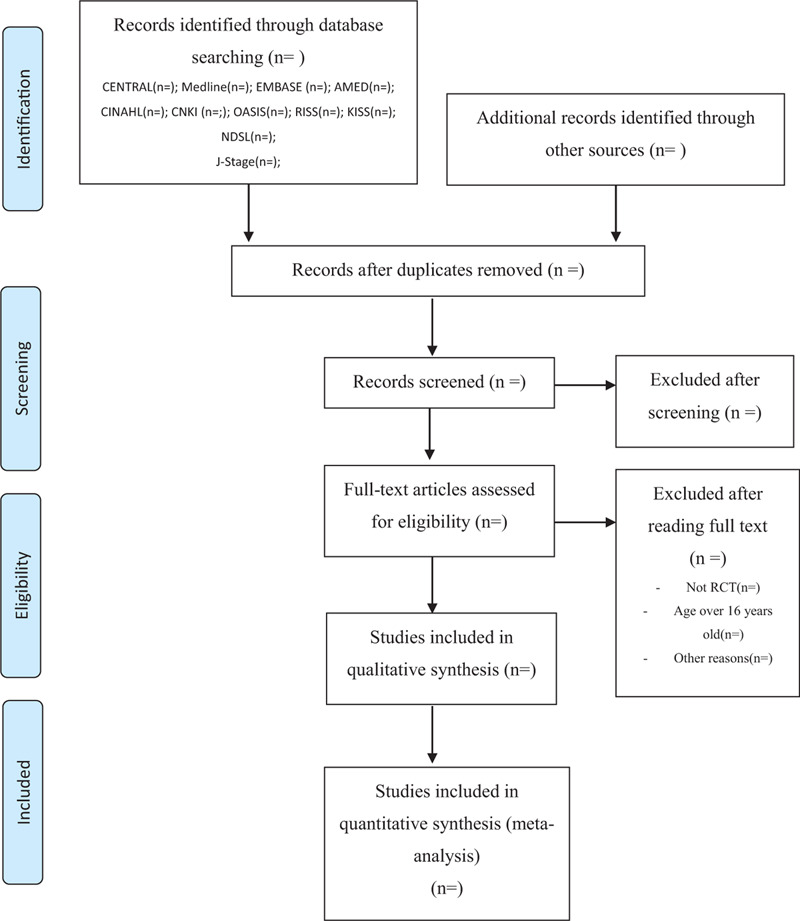
PRISMA flow chart of the search process. AMED = Allied and Complementary Medicine Database, CENTRAL = Cochrane Central of Controlled TRIALS, CINAHL = Cumulative Index to Nursing and Allied Health Literature, CNKI = China National Knowledge Infrastructure, EMBASE = Excerpta Medica dataBASE, J-Stage = Japan Science Technology Information Aggregator, Electronic, KISS = Korean Studies Information Service System, NDSL = National Digital Science Library, OASIS = Oriental Medicine Advanced Searching Integrated System, PRISMA = preferred reporting items for systematic reviews and meta-analysis, RCT = randomized clinical trials, RISS = Research Information Service System.

We will only include studies on pediatric patients with LRTIs aged 0 to 16 years, and the pediatric age will be categorized following the US FDA definition.^[21]^ The patients should have clinically and radiologically diagnosed acute LRTIs such as pneumonia, bronchitis, and bronchiolitis. No restriction will be imposed on the cause, viral or bacterial infections; however, studies on aspiration pneumonia will be excluded. We will also exclude studies on chronic LRTIs (persisting for more than 3 weeks) or chronic respiratory diseases such as asthma, bronchiectasis, and tuberculosis. A detailed search will be conducted according to the preferred reporting items for systematic reviews and meta-analysis guidelines (Fig. 1).

#### 2.2.3 Types of interventions

We will only include studies using MHGT as an experimental intervention. Only oral administration will be considered. All kinds of MHGT formulations will be allowed (eg, decoction, tablets, capsules, pills, powders, and extracts). As a control intervention, the studies should have included placebo or western medicines such as antibiotics.

### 2.3 Outcome measures

One of the following outcome measures will be used.

#### 2.3.1 Primary outcomes

(1) Total effective rate: This is the number of patients who showed an improvement in symptoms among the total subjects. Most Chinese controlled trials generally use the total effective rate when they report outcome measures in their studies (eg, “cured,” “markedly improved,” “improved,” “slightly better,” and “no effect”). It shows the effectiveness of the intervention. We will interpret the effective rate as reported by the included RCTs.
(2) Symptom disappearance time (fever, cough, change in lung sound, and X-ray): Fever, pulmonary abnormalities, cough, and chest X-ray lesion findings are typical symptoms of LRTIs, and the disappearance of these symptoms is an important indicator to determine whether a patient has recovered from respiratory disease.
(3) Inflammatory markers (eg, C-Reactive Protein, IL-6, and IL-10).

#### 2.3.2 Secondary outcomes

(1) Adverse events
(2) Complication rate of LRTIs

### 2.4 Data extraction and quality assessment

All articles will be read fully by 2 independent reviewers (ARJ and HMS), and data will be extracted according to pre-defined criteria. Quality assessment will be performed by the 2 reviewers using the Cochrane Handbook (V.5.1.011).^[22]^ The quality of the RCTs will be evaluated according to the following 7 categories:

(1) allocation concealment,
(2) random sequence generation,
(3) blinding of participants and personnel,
(4) blinding of outcome measures,
(5) incomplete outcome data,
(6) selective reporting, and
(7) other risk of biases.

We will report the risk of bias of each included RCT as “low risk,” “unclear risk,” and “high risk.” In case of a disagreement between the 2 reviewers, it will be resolved through discussion, if needed, by seeking the opinion of other reviewers (YSB and LHY).

### 2.5 Data synthesis

An analysis to compare the results of the intervention and control groups will be conducted. A quantitative analysis of a single measurement of each outcome will be conducted using Review Manager version 5.3. To summarize the effects of the herbal medicine on each outcome, we will abstract the risk estimates (relative risk) for dichotomous data and mean differences or standardized mean differences for continuous data with 95% confidence intervals.

We will calculate the relative risk, standardized mean differences, or weight mean difference with 95% confidence intervals using Review Manager Version 5.3 software for Windows (Cochrane, London, UK).

### 2.6 Units of analysis issues

All parallel and crossover design studies will be included in this review. In case of crossover studies, we will analyze only the first treatment period data of the included studies. All RCTs with multiple control groups will be analyzed if they meet our inclusion criteria.

### 2.7 Dealing with missing data

In the case of missing or insufficient data, we will try our best to contact the authors of the relevant studies via e-mail or telephone. If we fail to obtain the missing data, the data will not be analyzed. The potential effects of the missing data will be discussed in this review.

### 2.8 Assessment heterogeneity

We will use the *χ*^2^ test and *I*^2^ value to analyze heterogeneity in the study results. An *I*^2^ value of more than 50% will be considered to indicate a significant heterogeneity between the studies. In the meta-analysis, random-effect models will be used for pooling data across studies when the heterogeneity between studies is significant, whereas a fixed-model will be used when the heterogeneity is not significant. For a more comprehensive understanding of the meta-analysis, a subgroup or sensitivity analysis will be additionally conducted.

### 2.9 Assessment of reporting bias

To evaluate reporting biases, we will use a funnel plot using Cochrane software if the number of included studies is sufficient (>10 studies). If there is no publication bias, the funnel plot will be symmetrical, whereas if there is publication bias, the funnel plot will be asymmetrical. In case of existing publication bias, we will try to discuss possible reasons.

### 2.10 Subgroup analysis

If there is a significant heterogeneity (*I*^2^ > 50%) and the necessary data are available, we will conduct a subgroup analysis of the following: treatment period, cause of LRTIs (eg, viral and bacterial infections), and MHGT formulation (eg, decoction and granules).

### 2.11 Sensitivity analysis

To confirm the robustness of the meta-analysis, we will identify whether the results change with the exclusion of poor quality studies, missing values, and outliers.^[22]^

### 2.12 Quality of evidence

To evaluate the quality of evidence for each outcome, we will use the Grading of Recommendations Assessment, Development, and Evaluation approach.^[23]^ The quality of evidence will be classified using a 4-point scale: high, moderate, low, and very low.

### 2.13 Ethical approval

Ethical approval is not necessary because this study will be based on published research.

## 3 Discussion

MHGT is a representative prescription for wind-heat syndrome in the respiratory tract. It has been widely used for treating patients with LRTIs in Asia; therefore, MHGT is a good therapeutic option for patients with LRTIs. Studies have shown that MHGT exerts anti-inflammatory effects in lung injury by regulating the NF-κB pathway. Furthermore, clinical trials on MHGT for LRTIs have indicated its efficacy against LRTIs. However, its efficacy has not been evaluated systematically. The aim of this study is to systematically evaluate the efficacy and safety of MHGT in patients with LRTIs. The severity of the disease and the alteration in prescriptions could lead to a risk of heterogeneity. The results of this study will assist clinicians in the prescription of MHGT for LRTI treatment.

## Author contributions

YSB and JAR conceptualized and designed the study. JAR, YSB, and LHY developed the search strategy. JAR and HMS drafted the manuscript. HMS and LHY interpreted and revised this article. All authors read and approved the final manuscript.

## Supplementary Material

Supplemental Digital Content
