# Supplementary material for: Mahaenggamseok-tang, a herbal medicine, for lower respiratory tract infections in pediatric patients: A protocol for systematic review and meta-analysis
Source: Medicine (Baltimore). 2020 Sep 4;99(36):e21951. doi: 10.1097/MD.0000000000021951 (PMC7478659; doi:10.1097/MD.0000000000021951)
Supplement: Supplemental Digital Content [file medi-99-e21951-s001.docx]

**Appendix A. Search strategy used in the English databases.**

#1. Mahaenggamseok-tang [Mesh Terms]

#2. (Ma-xing-shi-gan-tang* or (MXSGT decoction) or (Maxingshigan*) or (Ma Xing Shi Gan*) or

(Maxing Ganshi*) or (Makyokansekito*) [tiab]

#3. #1 OR #2

#4. Lower Respiratory Tract Infection [Mesh Terms]

#5. (bronchitis or (bronchiolitis) or (pneumonia) or (broncho^*^) or (bronchial^*^) or (lung inflammation)) [tiab]

#6. #4 OR #5.

#7. Randomized controlled trial [pt]

#8. Controlled clinical trial [pt]

#9. Randomized [tiab]

#10. Randomly [tiab]

#11. Trial [ti]

#12. #7 OR #8 OR #9 OR #10 OR #11

#13. #3 AND #6 AND #12

**Appendix B. Search strategy used in the Chinese databases.**

#1. 麻杏甘石汤

#2. 麻杏石甘汤

#3. #1 OR #2

#4. 喘嗽

#5. 肺炎

#6. 小儿肺炎

#5. 小儿喘嗽

#6. 支气管肺炎

#7. 周围神经毒性

#8. 小儿支气管肺炎

#9. 小儿支原体肺炎

#10. #4 OR #5 OR #6 R #7 OR #8 OR #9

#11. #3 AND #10
